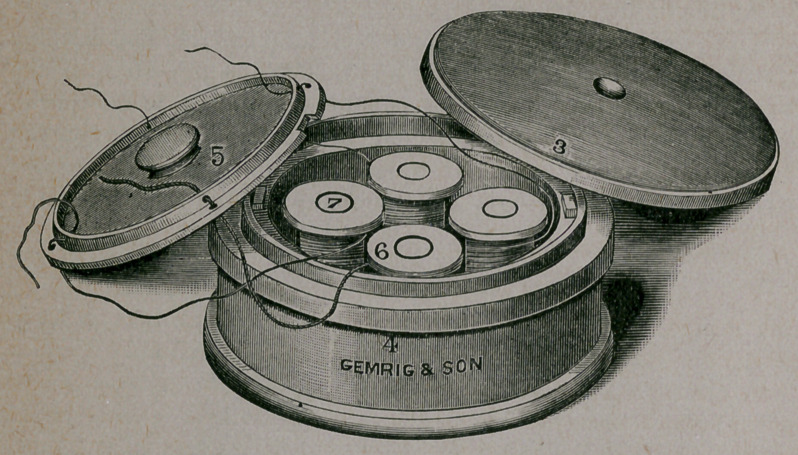# Antiseptic Ligature Box

**Published:** 1889-09

**Authors:** H. M. Weeks


### New Instruments.

AT THE regular meeting of the Philadelphia Obstetrical Society, held June 7, 1889, Dr. H. M. WEEKS, of Trenton, N. J., exhibited An Antiseptic Ligature Box.

This box is presented to the profession for preserving and carrying ligatures that have been prepared and rendered aseptic or antiseptic, enabling the operator to cut his ligatures and sutures, at the time of operating, without danger of soiling or infecting the portion not required for immediate use. It is made of a fine quality of earthenware, thus securing strength and durability; at the same time, it is light, compact, ornamental; and last, but not least, it can be furnished at a price that will enable every one practising surgery to provide himself with one or more. The box can be had in any color desired, or with any decoration the consumer may wish.

The accompanying cut represents the different parts as follows: The box is round, four inches in diameter and two inches high, with an outside cover, No. 3, that is held in position by a neat clamp, No. 1, which, when adjusted, is prevented from slipping by a slot on either side of the band or flange at the top of the box, the screw holding the cover tightly down upon the rubber washer, No. 3, which encircles the top, and renders the box absolutely air and fluid tight, so that the ligatures can be carried constantly in any solution desired without danger of leakage.

The inner cover, No. 5, is a flat disk with a slot cut in the edge to allow it to be placed in position, and held by two small catches placed on opposite sides of the box; the small knob in the center serves to turn and place and remove the cover. There are four holes perforating this cover for the four sizes of silk generally used; and half an inch from the edge of the cover there is a raised band, also perforated, for the silk to pass, thus making it impossible for the end of the ligature to drop back into the box when cut. This cover rests upon a ledge, and is left in place except when necessary to fill the reels or spools with silk, or the box with solution.

The reels or spools, No. 6, four in number, stand upright, and are held in position by separate spindles, No. 7. The whole box is highly glazed ; there is no metal or anything that can be acted upon by any solutions, and the material from which it is made can be subjected to any amonnt of heat, either dry or by boiling. It can be taken apart in a very few seconds, and every part thoroughly cleansed.

Should any of the parts break, they can be replaced, as they are interchangeable.

They may be obtained from J. H. Gemrig & Son, 109 South Eighth street, Philadelphia.

---

MERCK'S BULLETIN for June, 1889, contains a table of especial value, giving, as it does, the *Maximal Doses*—by grains and grammes—of 113 of the newer remedies. For many of these remedies no reliable table of dose limits has hitherto been published in this country, and therefore its importance will at once be recognized. For the benefit of those who may desire to possess this table we will say that it may be obtained by addressing "Merck's Bulletin, No. 37 William street, New York." The Bulletin is published once a month, and gives the latest information concerning all the newer remedies as they appear. It is a useful publication, and well worth the small subscription price of one dollar a year.

## Figures and Tables

**Figure f1:**
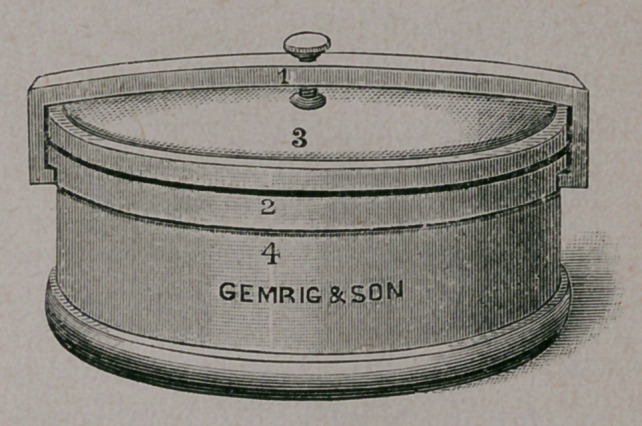


**Figure f2:**